# HBV Infection Status and the Risk of Cholangiocarcinoma in Asia: A Meta-Analysis

**DOI:** 10.1155/2016/3417976

**Published:** 2016-11-23

**Authors:** Hao Zhang, Biqing Zhu, He Zhang, Jianxin Liang, Wenting Zeng

**Affiliations:** ^1^Department of Infectious Diseases, The First Affiliated Hospital of Guangzhou Medical University, Guangzhou Medical University, Yanjiang Road 151, Guangzhou 510120, China; ^2^Department of Laboratory Medicine, Dongguan Hospital of Traditional Chinese Medicine, Guangzhou University of Chinese Medicine, Songshan Lake Avenue 22, Dongcheng District, Dongguan 523000, China

## Abstract

*Background*. The inconsistent finding was between hepatitis B virus (HBV) infections and cholangiocarcinoma (CCA). This meta-analysis is to explore this relationship in Asia.* Methods*. A literature search was performed using PubMed, Web of Science, and Cochrane Library to October 30, 2015. Pooled incidence rate and OR with 95% CI were calculated using STATA 11.0.* Results*. Thirty-nine studies were included. The pooled incidence rate of CCA patients with HBV infection was 31% (95% CI 22%–39%). The pooled OR showed increased risk of CCA incidence with HBV infection (OR = 2.72, 95% CI 1.90–3.88), especially in ICC (OR = 3.184, 95% CI 2.356–4.302), while it showed no risk in ECC (OR = 1.407, 95% CI 0.925–2.141). Also, the pooled OR showed increased risk of ICC and ECC incidence (OR = 6.857, 95% CI 4.421–10.633 and OR = 1.740, 95% CI 1.260–2.404) in patients with HBsAg+/HBcAb+. The pooled OR showed increased risk of ICC incidence (OR = 1.410, 95% CI 1.095–1.816) in patients with HBsAg−/HBcAb+.* Conclusion*. It is suggested that HBV infection is associated with an increased risk of CCA in Asia. Two HBV infection models (HBsAg+/HBcAb+ and HBsAg−/HBcAb+) increase the risk of CCA, and patients with HBsAg−/HBcAb+ also had a risk of ICC. This trial is registered with PROSPERO CRD42015029264.

## 1. Introduction

Cholangiocarcinoma (CCA) is the second commonest primary liver tumor worldwide after hepatocellular carcinoma (HCC) [1]. In the World Health Organization (WHO), CCA is classified as intrahepatic cholangiocarcinoma (ICC) or extrahepatic cholangiocarcinoma (ECC). It accounts for 3% of all gastrointestinal tumors [2]. During the past 3 decades, the overall incidence rate of CCA has appeared to have significantly increased [1]. Hepatobiliary malignancies account for 13% of cancer-related deaths and 10%–20% of these are attributable to CCA worldwide [3]. Currently, CCA has not been extensively investigated, possibly as a result of its presumptive rarity and worrisome prognosis at the time of diagnosis. It has been shown that CCA is characterized by a low survival rate with a median survival of less than 24 months after diagnosis [4].

Defining the risk factors for CCA benefits the search for better ways to prevent the occurrence of this disease. To date, it has been shown that viral infection (mainly hepatitis B and hepatitis C infection), gallstone formation, choledochoenteric anastomosis, oil sands carcinogenicity, chemicals and radiations exposure, biliary system development abnormalities, and so on are the major risk factors for CCA [5]. However, data from low hepatitis B (HBV) epidemic area and HBV infection is not a risk factor for CCA [6]. Thus, we conducted this meta-analysis for evaluating the association between HBV infection and CCA (including ICC and ECC) in Asian countries which are high HBV epidemic areas.

Also, two main treatment options in hepatitis B are available: interferon and nucleos(t)ide analogue (NUCs). There are five NUCs currently approved for HBV treatment: lamivudine (LAM), adefovir (ADV), telbivudine (LdT), entecavir (ETV), and tenofovir disoproxil fumarate (TDF). Treatment with TDF or ETV is more effective due to a high antiviral potency and has a high barrier to resistance reducing the risk of drug resistance and treatment failure [7]. So, HBV DNA could be inhibited during treatment. Even, some patients could achieve serological conversion of HBsAg (HBsAg−/HBcAb+) whereas, for patients with curative resection of HBV-related HCC, positive HBcAb is associated with higher risk of early recurrence and poorer survival [8] and hepatitis B reactivation in HBsAg−/HBcAb+ patients receiving rituximab for lymphoma [9]. According to those, the risk of cancer was still existing in patients with HBsAg−/HBcAb+. Thus, this meta-analysis was also conducted for the association between HBV infection model and CCA risk.

## 2. Methods

### 2.1. Search Strategy

We have registered this trial with PROSPERO (http://www.crd.york.ac.uk/PROSPERO/) (trial number: CRD42015029264). Our study was performed according to the recommendations of the Moose [10] (see Supplementary Table  1 of the Supplementary Material available online at http://dx.doi.org/10.1155/2016/3417976). PubMed was searched combining the terms of (“hepatitis B” OR HBV OR CHB) AND (“bile duct neoplasms” OR cholangiocarcinoma).

### 2.2. Inclusion and Exclusion Criteria

For estimating the incidence rate of CCA, ICC, or ECC, the studies were included if they meet the criteria as follows: (i) studies only containing the cancer group; (ii) exposure to HBV infection; (iii) providing enough information for calculating the incidence rate; and (iv) the number of cases being not less than 50.

For estimating the OR of CCA, ICC, or ECC, the studies were included if they meet the criteria as follows: (i) exposure to HBV infection; (ii) the outcome being CCA, ICC, or ECC incidence; (iii) providing risk estimates with 95% confidence interval (CI) or available information to calculate them; and (iv) published full-text report in English language.

Abstracts and reviews, letters, case reports, and studies that did not provide sufficient data to calculate the risk estimates were excluded. Two investigators (Hao Zhang and Biqing Zhu) independently selected studies, and any discrepancies were resolved by the third investigator (Wenting Zeng).

### 2.3. Quality Assessment

The quality of the included studies was assessed independently by two authors (Hao Zhang and Jianxin Liang) using the Newcastle-Ottawa Scale (NOS) [46]. The NOS is for observed studies and consists of 3 parameters of quality: selection, comparability, and exposure/outcome assessment. The NOS assigns a maximum of 4 points for selection, 2 for comparability, and 3 for exposure or outcome. We assigned NOS scores of 1–3, 4–6, and 7–9 for low-, intermediate-, and high-quality studies. Discrepancies were settled by consensus after joint reevaluation of the original studies with the third author (Jianxin Liang).

### 2.4. Data Extraction

The following information was extracted from each study: first author, publication time, the sample size, country, number of exposures in cases and controls; risk estimate; and 95% CI. The data were collected independently by two investigators (Hao Zhang and Biqing Zhu). When the literature citations were controversial, these investigators discussed them and reached a consensus on inclusion or exclusion.

### 2.5. Data Analysis

The overincidence rate of CCA, ICC, and ECC with HBV infection was calculated by effect size (ES) and the corresponding 95% confidence interval (95% CI). The risk of HBV infection outcomes was estimated by odds ratio (OR) with the corresponding 95% CI. It was considered statistically significant when *P* < 0.05. In the forest plots, OR > 1 represented a risk effect and OR < 1 represented a protective effect. Statistical heterogeneity of results was appraised using a Chi-Square based *Q* test and *I* ^2^ statistic. Only when the analysis fullfilled both *P* > 0.10 and *I* ^2^ < 50%, the heterogeneity was considered not significant. The fixed-effects model was used when literature heterogeneity did not exist; otherwise, the random-effects model was employed. Sensitivity analysis was conducted by modification of the inclusion criteria of this meta-analysis. The pooled proportion of vertical transmission of toxoplasmosis was calculated by STATA 11.0 software (Stata Corporation, College Station, TX, USA) and the publication bias was considered significant when *P* value was less than 0.05 in either Begg's test or Egger's test.

## 3. Results

### 3.1. Selection of Studies

**Figure 1:**
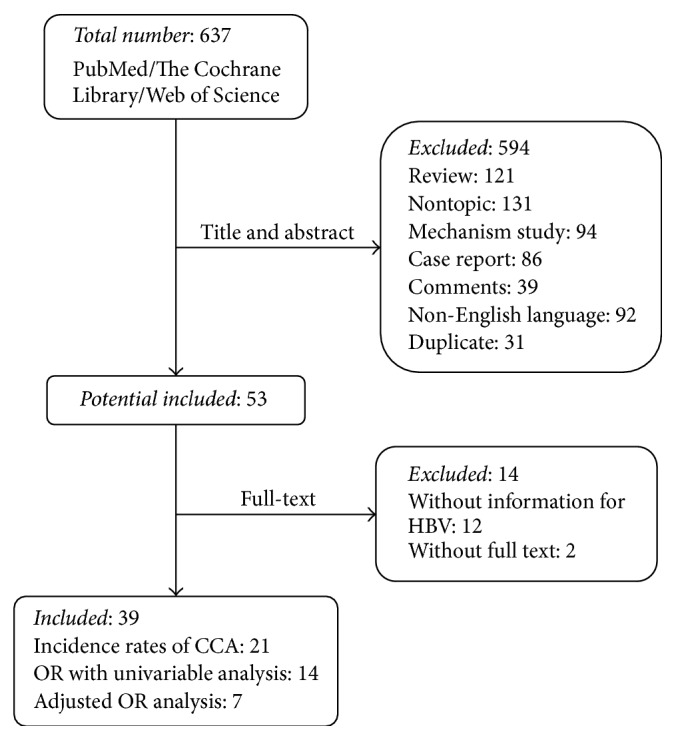
Flow diagram of selection and disposition of studies.

A flow diagram of the selection of the studies is shown in Figure 1. A total of 39 studies with CCA matched the inclusion criteria in this meta-analysis, including 21 studies [11–31] for estimating the incidence rates of CCA, 14 studies [32–45] for unadjusted OR, and 7 studies [32, 33, 36, 39, 41, 42, 44] for adjusted OR analysis (Tables 1 and 2). Among the 39 studies, 26 studies were for ICC, including 13 studies [11–14, 16, 17, 19, 22, 23, 25, 26, 29, 31] for estimating the incidence rates of ICC, 11 studies [32, 34–36, 39–42, 44, 45, 47] for unadjusted OR analysis, and 7 studies [32, 35, 36, 39, 41, 42, 44] for adjusted OR analysis (Tables 3 and 4). Nine studies were for ECC, including 3 studies [11, 15, 20] for estimating the incidence rates of ECC, 5 studies [32, 35, 40, 43, 47] for unadjusted OR analysis, and 1 study [35] for adjusted OR analysis (Tables 4 and 5). The main features of included studies were shown in Tables 1 [Table tab2] [Table tab3] [Table tab4]–5. Among the included studies, 4 studies are from Korea [24, 29, 32, 43], 4 from Japan [11, 28, 30, 45], 3 from Thailand [18, 30, 38], and 24 from China [12–17, 19–23, 25–27, 33–37, 39–43]. For analyzing the association between HBV infection and CCA risk, twelve studies (75%) were of high quality and the other four (25%) were acceptable based on the NOS scores.

### 3.2. HBV Infection and the Risk of CCA

**Figure 2:**
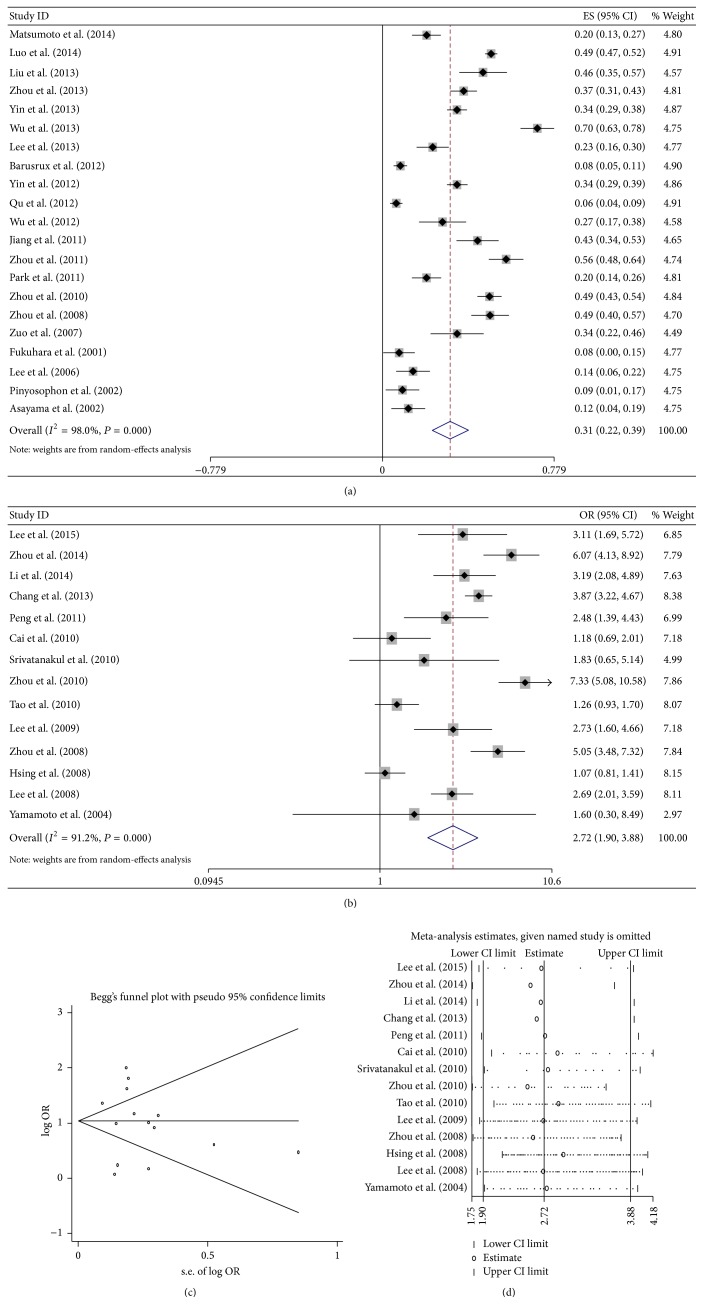
Meta-analysis of the association between HBV infection and CCA. (a) Pooled incidence of CCA in Asia; (b) forest plots of the association between HBV infection and CCA with unadjusted OR; (c) sensitivity analysis of the association between HBV infection and CCA; and (d) forest plots of the association between HBV infection and CCA with adjusted OR.

For pooled analysis of incidence rates of CCA with HBV infection, 21 studies with 6 253 participants were included [11–31]. All these studies were from Asia, including 3 from Japan, 2 from Thailand, one from Korea, and 15 from China. The overall pooled incidence rates were 31% (95% CI 22%–39%), calculated with the random-effects model (*P* < 0.001, *I* ^2^ = 98.0%, Figure 2(a)). In China, the pooled incidence rates were 37.6% (95% CI 26.8%–48.3%, *P* < 0.001), calculated with the random-effects model (*P* < 0.001, *I* ^2^ = 98.5%).

For pooled analysis of unadjusted OR of CCA with HBV infection, 14 studies with 24 337 participants including controls were analyzed [32–45]. The meta-analysis in a random-effects model (*P* < 0.001, *I* ^2^ = 91.2%) found statistically significant increased risk of CCA incidence with HBV infection (OR = 2.72, 95% CI 1.90–3.88, *P* < 0.001, Figure 2(b)). No publication bias was observed by Begg's test (*P* = 0.443) and Egger's test (*P* = 0.774). The sensitivity analysis showed that the result that HBV infection was a high risk for patients progressing into CCA was not changed (Figure 2(c)).

For pooled analysis of adjusted OR of CCA with HBV infection, 7 studies with 6,883 participants including controls were analyzed [32, 33, 36, 39, 41, 42, 44]. The meta-analysis in a random-effects model (*P* < 0.001, *I* ^2^ = 92.2%) also found statistically significant increased risk of CCA incidence with HBV infection (OR = 5.903, 95% CI 3.110–11.207, *P* < 0.001, Figure 2(d)).

### 3.3. HBV Infection and the Risk of ICC

**Figure 3:**
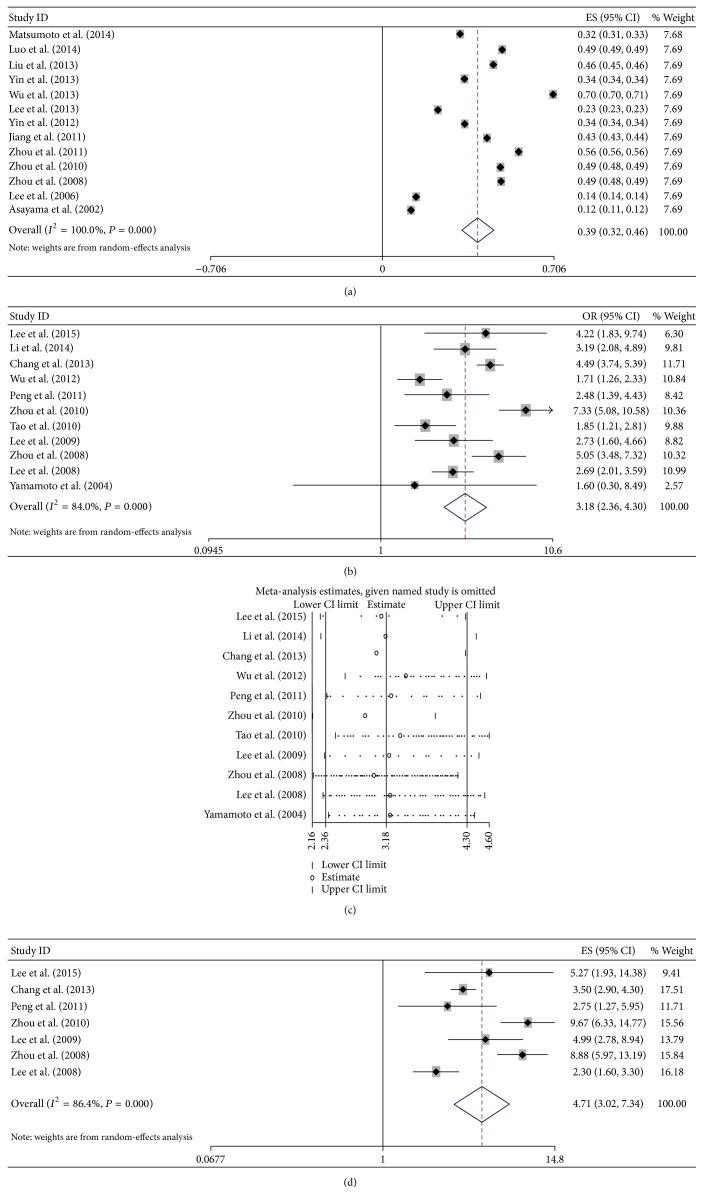
Meta-analysis of the association between HBV infection and ICC. (a) Pooled incidence of ICC in Asia; (b) forest plots of the association between HBV infection and ICC with unadjusted OR; (c) sensitivity analysis of the association between HBV infection and ICC; and (d) forest plots of the association between HBV infection and ICC with adjusted OR.

For pooled analysis of incidence rates of ICC with HBV infection, 13 studies with 4,871 participants were included [11–14, 16, 17, 19, 22, 23, 25, 26, 29, 31]. All these studies were from Asia, including 2 were from Japan, 1 from Korea, and 10 from China. The overall pooled incidence rates were 39% (95% CI 32%–46%, *P* < 0.001), calculated with the random-effects model (*P* < 0.001, *I* ^2^ = 100%, Figure 3(a)).

For pooled analysis of unadjusted OR of ICC with HBV infection, 11 studies with 22 924 participants including controls were analyzed [32, 34–36, 39–42, 44, 45, 47]. The meta-analysis in a random-effects model (*P* < 0.001, *I* ^2^ = 84.0%) found statistically significant increased risk of ICC incidence with HBV infection (OR = 3.184, 95% CI 2.356–4.302, *P* < 0.001, Figure 3(b)). No publication bias was observed by Begg's test (*P* = 0.755) and Egger's test (*P* = 0.428). The sensitivity analysis showed that the result that HBV infection was a high risk for patients progressing into ICC was not changed (Figure 3(c)).

For pooled analysis of adjusted OR of ICC with HBV infection, 7 studies with 20,564 participants including controls were analyzed [32, 35, 36, 39, 41, 42, 44]. The meta-analysis in a random-effects model (*P* < 0.001, *I* ^2^ = 86.4%) also found statistically significant increased risk of ICC incidence with HBV infection (OR = 4.709, 95% CI 3.020–7.341, *P* < 0.001, Figure 3(d)).

### 3.4. HBV Infection and the Risk of ECC

**Figure 4:**
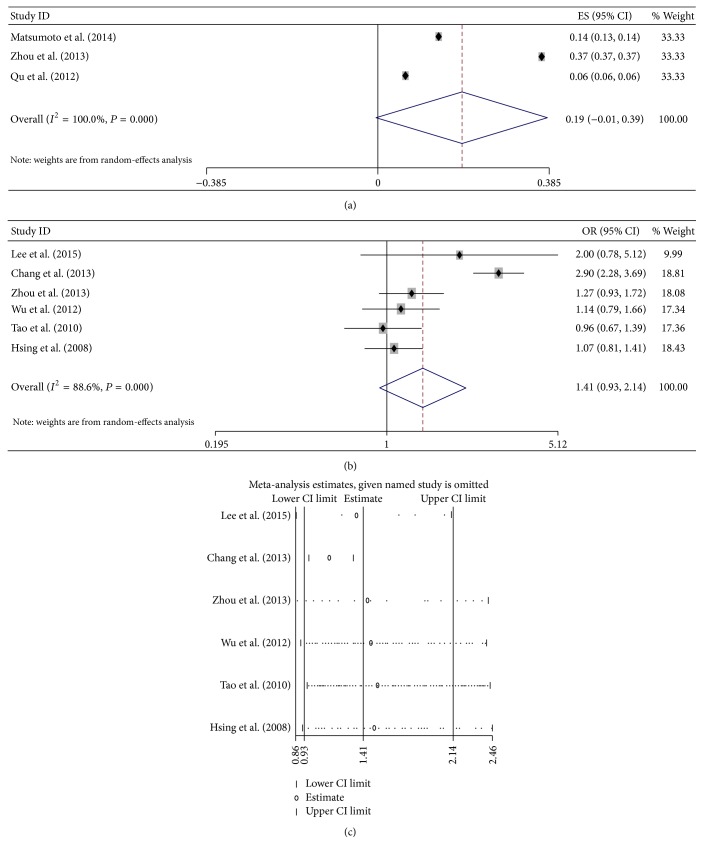
Meta-analysis of the association between HBV infection and ECC. (a) Pooled incidence of ECC in Asia; (b) forest plots of the association between HBV infection and ECC with unadjusted OR; and (c) sensitivity analysis of the association between HBV infection and ECC.

For pooled analysis of incidence rates of ECC with HBV infection, 2 studies with 483 participants were included [11, 20]. These studies were from Japan and China. The overall pooled incidence rates were 18.9% (95% CI −0.7%–38.5%, *P* = 0.059), calculated with the random-effects model (*P* < 0.001, *I* ^2^ = 100%, Figure 4(a)).

For pooled analysis of unadjusted OR of ECC with HBV infection, 6 studies with 14 517 participants including controls were analyzed [15, 32, 35, 40, 43, 47]. The meta-analysis in a random-effects model (*P* < 0.001, *I* ^2^ = 88.6%) found no statistically significant increased risk of ECC incidence with HBV infection (OR = 1.407, 95% CI 0.925–2.141, *P* = 0.110, Figure 4(b)). No publication bias was observed by Begg's test (*P* = 1.0) and Egger's test (*P* = 0.579). The sensitivity analysis showed that the result that HBV infection was not a high risk for patients progressing into ECC was not changed (Figure 4(c)).

However, the adjusted OR of ECC with HBV infection found statistically significant increased risk of ECC incidence with HBV infection (OR = 2.6, 95% CI 2.0–3.4), which was only in one study [35].

### 3.5. HBV Infection Status and the Risk of Cancer

**Figure 5:**
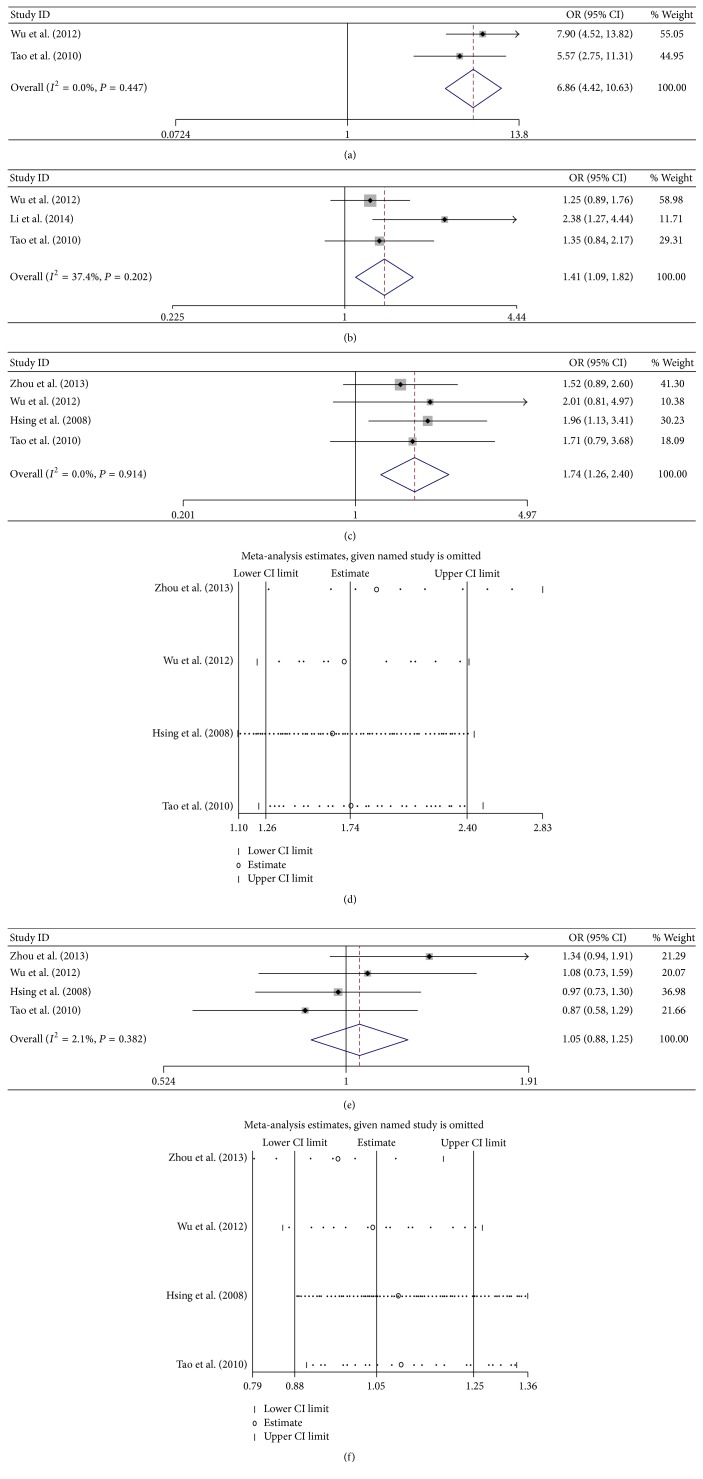
Meta-analysis of the association between HBV infection models and cancer. (a) Forest plots of the association between HBsAg+/HBcAb+ and ICC; (b) forest plots of the association between HBsAg−/HBcAb+ and ICC; (c) forest plots of the association between HBsAg+/HBcAb+ and ECC; (d) sensitivity analysis of the association between HBsAg+/HBcAb+ and ECC; (e) forest plots of the association between HBsAg−/HBcAb+ and ICC; and (f) sensitivity analysis of the association between HBsAg−/HBcAb+ and ECC.

Two HBV infection models, which were HBsAg+/HBcAb+ and HBsAg−/HBcAb+, were included in this meta-analysis. Patients with HBsAg+/HBcAb+ showed a high risk of ICC (OR = 6.857, 95% CI 4.421–10.633) with calculating in a fix-effects model (*P* = 0.447, *I* ^2^ = 0%, Figure 5(a)). As only two studies were analyzed, the publication bias and sensitivity analysis cannot be performed. The risk of ICC was increased in patients with HBsAg−/HBcAb+ (OR = 1.410, 95% CI 1.095–1.816) with calculating in a fix-effects model (*P* = 0.202, *I* ^2^ = 37.4%, Figure 5(b)). No publication bias was observed by Begg's test (*P* = 0.96) and Egger's test (*P* = 0.270). As only three studies were analyzed, the sensitivity analysis cannot be performed.

Patients with HBsAg+/HBcAb+ showed a high risk of ECC (OR = 1.740, 95% CI 1.260–2.404) with calculating in a fix-effects model (*P* = 0.914, *I* ^2^ = 0%, Figure 5(c)). No publication bias was observed by Begg's test (*P* = 0.734) and Egger's test (*P* = 0.627). The sensitivity analysis showed that the result that HBV infection was a high risk for patients progressing into ECC was not changed (Figure 5(d)). The risk of ECC was not increased of patients with HBsAg−/HBcAb+ (OR = 1.049, 95% CI 0.881–1.249) with calculating in a fix-effects model (*P* = 0.382, *I* ^2^ = 2.1%, Figure 5(e)). No publication bias was observed by Begg's test (*P* = 0.734) and Egger's test (*P* = 0.993). As only three studies were analyzed, the sensitivity analysis cannot be performed. The sensitivity analysis showed that the result that HBV infection was not a high risk for patients progressing into ECC was not changed (Figure 5(f)).

## 4. Discussion

HBV is widely epidemic in Asian countries [48]. Previous study has concluded that HBV infection is associated with an increased risk of ICC [49]. This meta-analysis was employed to estimate the CCA incidence rate in HBV infection patients and the CCA risk of HBV infection models in Asia, including ICC and ECC.

In this study, HBV infection leading to CCA was about 31%, and this rate (39%) was also high in China. HBV infection, where pooled OR is 2.72 (95% CI 1.90–3.88) and adjusted OR is 5.903 (95% CI 3.110–11.207), is a risk factor of CCA. This phenomenon also occurred in the patients with ICC. As all we know, HBV is a strong risk factor for HCC. And hepatocytes and cholangiocytes have the same progenitor cell; therefore, it can be postulated that HBV can induce carcinogenesis in both hepatocytes and cholangiocytes by the same mechanism [50]. Furthermore, studies show that HBV has been suggested to be involved in the pathogenesis of ICC through the inflammatory process [51, 52], which further supports the potential role of HBV infection in the pathogenesis of cholangiocarcinoma. Also, recent clinical surveys in China have detected HBV DNA in the tissue species from bile duct cancer.

Interestingly, the risk of ICC was also excluded in patients with the model of HBsAg−/HBcAb+ that termed non-B non-C. The main mechanism may be that these patients were occult HBV infection. It is reported that even a history of HBV infection (positive HBcAb+ with HBsAg−) can be a risk factor for HCC [53]. HBcAb positive status may reflect occult HBV infection and could be associated with an increase in the risk of carcinogenesis [54]. Also, a recent study reported that positive HBcAb is associated with a higher risk of early intrahepatic recurrence and poorer relapse-free survival of HBV-related HCC patients after curative resection [8], which means that HBcAb may play a role in the HCC patients.

Although the incidence rate of ECC was not significantly different in HBV infection patients and HBV infection was not a risk of patients progressing into ECC, the HBV infection status was much different. Patients with HBsAg+/HBcAb+ showed a high risk of ECC, while patients with HBsAg−/HBcAb+ showed no risk of ECC. The mechanism may be associated with HBsAg status. Though few studies have explained the link between HBsAg and ECC, a recent report showed that HBsAg could stimulate proliferation and functional modification of hepatocytes via LEF-1 through the Wnt pathway at the premalignant stage of HCC [55]. Also, high HBsAg levels were associated with late recurrence (after 2 years) after curative resection in HBV-related HCC [56]. So, HBsAg might use a similar action to affect ECC.

In general, our meta-analysis results were similar to those recently published by others about this subject [57]. However, this meta-analysis has several weaknesses that should be considered. First, the included studies were all observational studies that might introduce selection and recall biases. Second, lots of results were based on the random-effect model analysis, which might not reduce the strength of evidence. Third, a small number of studies were used to analyze the HBV infection status and the risk of cancer. Finally, the included studies were mostly from China. Thus, in the future, more studies from other counties need to be performed to explain the link between HBV infection and risk of CCA.

## 5. Conclusion

This study suggests that HBV infection is associated with an increased risk of CCA in Asia. The two HBV infection models (HBsAg+/HBcAb+ and HBsAg−/HBcAb+) increase the risk of CCA, while the model of HBsAg−/HBcAb+ increases the risk of ICC. More studies, especially from low epidemic areas, were needed to explore the connection between HBV infection model and CCA risk.

## Supplementary Material

Table 1: A Proposed Reporting Checklist for Authors, Editors, and Reviewers of Meta-analyses

## Figures and Tables

**Table 1:** Incidence rate of studies of HBV infection and CCA.

Authors	Year	Country	HBV infection cases (*n*)	CCA cases (*n*)	Reference
Matsumoto et al.	2014	Japan	29	145	[11]
Luo et al.	2014	China	608	1233	[12]
Liu et al.	2013	China	37	81	[13]
Yin et al.	2013	China	139	411	[14]
Zhou et al.	2013	China	88	239	[15]
Wu et al.	2013	China	97	138	[16]
Lee et al.	2013	China (Taiwan)	29	127	[17]
Barusrux et al.	2012	Thailand	24	295	[18]
Yin et al.	2012	China	131	386	[19]
Qu et al.	2012	China	19	305	[20]
Wu et al.	2012	China	18	66	[21]
Jiang et al.	2011	China	44	102	[22]
Zhou et al.	2011	China	87	155	[23]
Park et al.	2011	Korea	32	160	[24]
Zhou et al.	2010	China	154	317	[25]
Zhou et al.	2008	China	64	131	[26]
Zuo et al.	2007	China	20	59	[27]
Fukuhara et al.	2001	Japan	4	53	[28]
Lee et al.	2006	Korea	11	79	[29]
Pinyosophon and Wiwanitkit	2002	Thailand	5	55	[30]
Asayama et al.	2002	Japan	8	68	[31]

**Table 2 tab2:** Characteristics of studies of HBV infection and CCA risk.

Authors	Year	Country	Cases (*n*)	Controls (*n*)	Control description	Adjusted OR (95% CI)	Study quality	Reference
Lee et al.	2015	Korea	276	552	Hospital-based control	4.12 (2.01–8.44)	7	[32]
Zhou et al.	2014	China	126	504	Hospital-based control	19.245 (13.260–27.931)	7	[33]
Li et al.	2014	China	183	549	Hospital-based control	NR	8	[34]
Chang et al.	2013	China (Taiwan)	5157	8716	Population-based control	NR	9	[35]
Peng et al.	2011	China	98	196	Hospital-based control	2.75 (1.27–5.95)	8	[36]
Cai et al.	2011	China	313	608	Hospital-based control	NR	7	[37]
Srivatanakul et al.	2010	Thailand	106	106	Population-based control	NR	6	[38]
Zhou et al.	2010	China	317	634	Hospital-based control	9.669 (6.329–14.77)	8	[39]
Tao et al.	2010	China	190	380	Hospital-based control	NR	8	[40]
Lee et al.	2009	China (Taiwan)	160	160	Hospital-based control	4.985 (2.775–8.945)	6	[41]
Zhou et al.	2008	China	312	438	Hospital-based control	8.876 (5.973–13.192)	6	[42]
Hsing et al.	2008	China	134	762	Population-based control	NR	8	[43]
Lee et al.	2008	Korea	622	2488	Hospital-based control	2.3 (1.6–3.3)	7	[44]
Yamamoto et al.	2004	Japan	50	200	Hospital-based control	NR	6	[45]

NR: not reported.

**Table 3 tab3:** Incidence rate of studies of HBV infection and ICC/ECC.

Authors	Year	Country	Cancer group	HBV infection cases (*n*)	CCA cases (*n*)	Reference
Matsumoto et al.	2014	Japan	ICC	16	50	[11]
Luo et al	2014	China	ICC	608	1233	[12]
Liu et al.	2013	China	ICC	37	81	[13]
Yin et al.	2013	China	ICC	139	411	[14]
Zhou et al.	2013	China	ECC	88	239	[15]
Wu et al.	2013	China	ICC	97	138	[16]
Lee et al.	2013	China (Taiwan)	ICC	29	127	[17]
Yin et al.	2012	China	ICC	131	386	[19]
Qu et al.	2012	China	ECC	19	305	[20]
Jiang et al.	2011	China	ICC	44	102	[22]
Zhou et al.	2011	China	ICC	87	155	[23]
Zhou et al.	2010	China	ICC	154	317	[25]
Zhou et al.	2008	China	ICC	64	131	[26]
Lee et al.	2006	Korea	ICC	11	79	[29]
Asayama et al.	2002	Japan	ICC	8	68	[31]

**Table 4 tab4:** Characteristics of studies of HBV infection and ICC risk.

Authors	Year	Country	Cases (*n*)	Controls (*n*)	Control description	Adjusted OR (95% CI)	Study quality	Reference
Lee et al.	2015	Korea	83	166	Hospital-based control	5.27 (1.93–14.38)	7	[32]
Li et al.	2014	China	183	549	Hospital-based control	NR	8	[34]
Chang et al.	2013	China (Taiwan)	2978	11912	Population-based control	3.5 (2.9–4.3)	9	[35]
Wu et al.	2012	China	102	835	Hospital-based control	NR	7	[47]
Peng et al.	2011	China	98	196	Hospital-based control	2.75 (1.27–5.95)	8	[36]
Zhou et al.	2010	China	317	634	Hospital-based control	9.669 (6.329–14.77)	8	[39]
Tao et al.	2010	China	61	380	Hospital-based control	NR	8	[40]
Lee et al.	2009	China (Taiwan)	160	160	Hospital-based control	4.985 (2.775–8.945)	6	[41]
Zhou et al.	2008	China	312	438	Hospital-based control	8.876 (5.973–13.192)	6	[42]
Lee et al.	2008	Korea	622	2488	Hospital-based control	2.3 (1.6–3.3)	7	[44]
Yamamoto et al.	2004	Japan	50	200	Hospital-based control	NR	6	[45]

NR: not reported.

**Table 5:** Characteristics of studies of HBV infection and ECC risk.

Authors	Year	Country	Cases (*n*)	Controls (*n*)	Control description	Adjusted OR (95% CI)	Study quality	Reference
Lee et al.	2015	Korea	193	386	Hospital-based control	NR	7	[32]
Chang et al.	2013	China (Taiwan)	2179	8716	Population-based control	2.6 (2.0–3.4)	9	[35]
Zhou et al.	2013	China	239	478	Hospital-based control	NR	7	[15]
Wu et al.	2012	China	86	835	Hospital-based control	NR	7	[47]
Tao et al.	2010	China	129	380	Hospital-based control	NR	8	[40]
Hsing et al.	2008	China	134	762	Population-based control	NR	8	[43]

NR: not reported.
