# Supplementary material for: HBV Infection Status and the Risk of Cholangiocarcinoma in Asia: A Meta-Analysis
Source: Biomed Res Int. 2016 Nov 23;2016:3417976. doi: 10.1155/2016/3417976 (PMC5141322; doi:10.1155/2016/3417976)
Supplement: Supplementary file 1 — Table 1: A Proposed Reporting Checklist for Authors, Editors, and Reviewers of Meta-analyses [file 3417976.f1.docx]

Table S1: A Proposed Reporting Checklist for Authors, Editors, and Reviewers of Meta-analyses

| Checklist item | Reported on page # |
| --- | --- |
| Reporting of background should include |  |
| Problem definition | 3 |
| Hypothesis statement | 3 |
| Description of study outcome(s) | 3-4 |
| Type of exposure or intervention used | 4 |
| Type of study designs used | 4 |
| Study population | 4 |
| Reporting of search strategy should include |  |
| Qualifications of searchers (eg, librarians and investigator | 4 |
| Search strategy, including time period included in the synthesis and keywords | 4 |
| Effort to include all available studies, including contact with authors | 4 |
| Databases and registries searched | 4 |
| Search software used, name and version, including special features used (eg, explosion) | 4 |
| Use of hand searching (eg, reference lists of obtained articles) | 5 |
| List of citations located and those excluded, including justification | 5 |
| Method of addressing articles published in languages other than English | 5 |
| Method of handling abstracts and unpublished studies | 5 |
| Description of any contact with authors | 5-6 |
| Reporting of methods should include |  |
| Description of relevance or appropriateness of studies assembled for assessing the hypothesis to be tested | 6 |
| Rationale for the selection and coding of data (eg, sound clinical principles or convenience) | 6 |
| Documentation of how data were classified and coded (eg, multiple raters, blinding, and  interrater reliability) | 6 |
| Assessment of confounding (eg, comparability of cases and controls in studies where  appropriate) | 6 |
| Assessment of study quality, including blinding of quality assessors; stratification or regression on possible predictors of study results | 6 |
| Assessment of heterogeneity | 6 |
| Description of statistical methods (eg, complete description of fixed or random effects models, justification of whether the chosen models account for predictors of study results,  dose-response models, or cumulative meta-analysis) in sufficient detail to be replicated | 6 |
| Provision of appropriate tables and graphics | 6-7 |
| Reporting of results should include |  |
| Graphic summarizing individual study estimates and overall estimate | 6-10 |
| Table giving descriptive information for each study included | 7 |
| Results of sensitivity testing (eg, subgroup analysis) | 6-10 |
| Indication of statistical uncertainty of findings | 6-10 |
| Reporting of discussion should include |  |
| Quantitative assessment of bias (eg, publication bias) | 10-12 |
| Justification for exclusion (eg, exclusion of non–English-language citations) | 10-12 |
| Assessment of quality of included studies | 10-12 |
| Reporting of conclusions should include |  |
| Consideration of alternative explanations for observed results | 12 |
| Generalization of the conclusions (ie, appropriate for the data presented and within the domain of the literature review) | 12 |
| Guidelines for future research | 12 |
| Disclosure of funding source | 12 |
